# Biomechanical analysis of different rotary files in varying root canal curvatures: a 3D finite element analysis study

**DOI:** 10.1186/s12903-026-08620-z

**Published:** 2026-05-29

**Authors:** Yathrib Mansy, Noha M. Elkersh, Raef Ahmed Sherif, Rewaa G. AboElHassan

**Affiliations:** 1 Division of Endodontics, Conservative Dentistry Department, Faculty of Dentistry, Alexandria University, Alexandria, Egypt; 2 Department of Oral Medicine, Periodontology, Oral diagnosis and Oral Radiology, Faculty of Dentistry, Alexandria University, Alexandria, Egypt; 3 Division of Fixed Prosthodontics, Conservative Dentistry Department, Faculty of Dentistry, Alexandria University, Alexandria, Egypt

**Keywords:** Finite element analysis, Root canal curvature, WaveOne gold, One curve, Nickel-titanium instruments

## Abstract

**Background:**

Proper biomechanical preparation of the root canal system is fundamental to the success of endodontic therapy. Curved root canals represent a major clinical challenge, increasing the risk of procedural errors such as canal transportation, ledge formation, and instrument separation. This study aimed to evaluate the stress distribution in two single-file rotary systems subjected to different canal curvatures using three-dimensional finite element analysis (FEA).

**Methods:**

Three-dimensional CAD models of root canals with curvatures of 2°, 30°, and 45° were created, along with models of WaveOne Gold and One Curve rotary files. Finite element simulations were performed to replicate clinical instrumentation conditions, and the generated stresses were evaluated using the von Mises stress criterion.

**Results:**

WaveOne Gold demonstrated maximum von Mises stress values of 375.42 MPa at 2°, 435.13 MPa at 30°, and 515.36 MPa at 45° curvature. Corresponding values for One Curve were 405.71 MPa, 472.87 MPa, and 565.17 MPa, respectively. Across all curvatures, WaveOne Gold exhibited lower stress values than One Curve.

**Conclusion:**

WaveOne Gold produced lower von Mises stress values than One Curve across all tested canal curvatures. Increasing canal curvature significantly increased stress levels in both file systems.

## Background

Successful root canal treatment involves debridement, disinfection, and obturation of the entire root canal system [1]. The purpose of root canal preparation is to create a continuously tapered shape with the lowest diameter at the apical foramen and the greatest at the orifice, allowing for efficient irrigation and filling [2]. It makes biomechanical preparation one of the major components of root canal treatment(RCT) [1]. 

This goal is frequently hard to attain while the dentist is dealing through the complicated interior anatomy of curved root canals [3]. Root canal curvature is utilised to determine RCT difficulty. The most frequent issues that arise when preparing curved root canals are step, root canal deviation, and instrument separation [4]. 

Before beginning treatment, radiographic tests are performed in clinical settings to assess the root canal curvature and RCT difficulties [5]. Schneider’s method divides the calculated root canal curvature into three levels: severely curved root canals (more than 30 degrees of curvature), moderately curved root canals (10–30 degrees of curvature), and straight root canals (0–10 degrees of curvature) [6]. 

Furthermore, the RCT difficulty is also influenced by the root canal curvature’s length and radius. At the same bending angle, the smaller the curved radius, the more difficult it is. As the distance between the apical stop and the root canal bend increases, the more difficult it is to bend the instruments at the curved point and, consequently, the greater the chance of instrument separation [5]. 

Endodontic files straighten curved canals, where the coronal region shifts towards the inner side of the curvature and the apical canal area enlarges close to the external surface of the canal warp. Therefore, it’s critical to stay away from aberrations that may result in iatrogenic injury during canal preparation, as this may unavoidably impact the treatment’s outcome [7]. 

Nickel–titanium (NiTi) rotary instruments have significantly improved the efficiency and safety of root canal preparation due to their superior flexibility and resistance to cyclic fatigue [8]. Among the contemporary systems, Wave One Gold (Dentsply Sirona, Balaguer, Switzerland) is a single-file system manufactured from a thermally treated gold-wire NiTi alloy and designed to operate in a reciprocating motion. This reciprocating movement consists of alternating clockwise and counterclockwise rotations, which helps reduce torsional stress and cyclic fatigue during canal preparation [9]. In contrast, One Curve (Micro-Mega, Besancon, France) is a single-file rotary system manufactured from C-wire heat-treated NiTi alloy and operates in continuous rotational motion. The instrument is designed to provide enhanced flexibility and improved shaping ability in curved canals. The differences in kinematics and metallurgical properties between these systems may influence their mechanical behavior and stress distribution during root canal instrumentation, particularly in canals with varying curvatures [10]. 

Inability to achieve apical third patency, asymmetrical dentin removal resulting in transportation, perforation, and tool fracture within the curved canals are among the procedural challenges that can compromise intraarticular infection treatment and result in a poor outcome [5]. 

When a structure experience loads, it produces distortion and stresses [11], these stresses cannot be directly measured, so, applying engineering expertise to dental field by utilising computational approaches has aided in the understanding of oral biomechanics [12]. 

Finite Element Analysis (FEA) takes dentistry to a new era [11]. In FEA, a specific physical system’s behaviour is mathematically simulated. A continuous structure is separated into various components that are joined by nodes while retaining the original structure’s characteristics [10, 13]. Material characteristics like Young’s modulus and Poisson ratio can be used in computer-generated assessments to explain a structure’s mechanical behaviour [14]. 

A structural analysis like this enables calculating the stress and strain brought on by pressure, heat change, external forces, and other factors [15]. Since it is impossible to quantify human tissue’s stress and strain in response to an external force, this approach is quite helpful for assessing the biomaterial’s mechanical properties in human tissues which are difficult to study directly on human subjects due to ethical considerations in conducting the research [16]. 

Considering the benefits of FEA and the significance of curvature of the canal in endodontics, the present research was commenced isolating the canal curvature as a single factor and investigated its effect on the amount of stress produced on the files, thus, the solitary role of canal curvature in stress generation understood well.

The aim of this work was to use FEA to assess the effect of different degrees of canal curvature on the stresses falling on two different single rotary files. The null hypothesis of the current study was that no differences in stress values would be recorded by the two rotary single files with different canal curvatures.

## Materials and methods

The present in vitro study was piloted in the department of endodontics in the faculty of dentistry, Alexandria University under ethical approval number of 950-8/2024-IORG 0008839.

### Construction of rotary files system

**Fig. 1:**
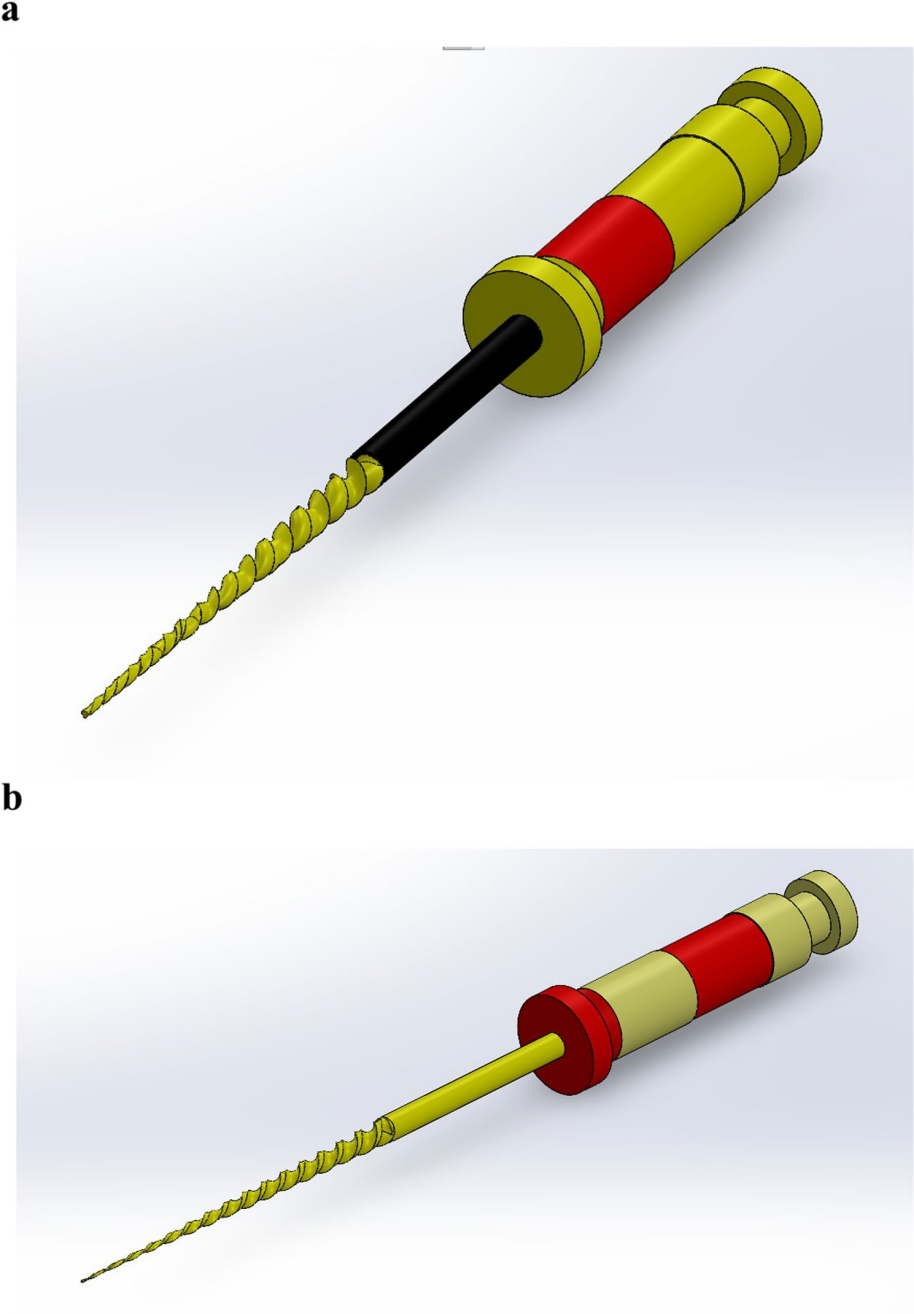
Three-dimensional (3D) Finite Element Model of rotary file. **a **Wave One Gold file, **b ** One Curve file

Cross-sectional shape of Wave One Gold (Dentsply, Maillefer, Switzerland) and One Curve (Micro-Mega, Besancon, Cedex, France) files were recorded using commercial file information like tapering of the files and the pitch using the Solid Work program, and the designs were executed in Solid Works software package 2020 by an engineer with computer aided design (CAD) knowledge to accurately design two rotary endodontic files (Fig. 1)**.**

**Fig. 2:**
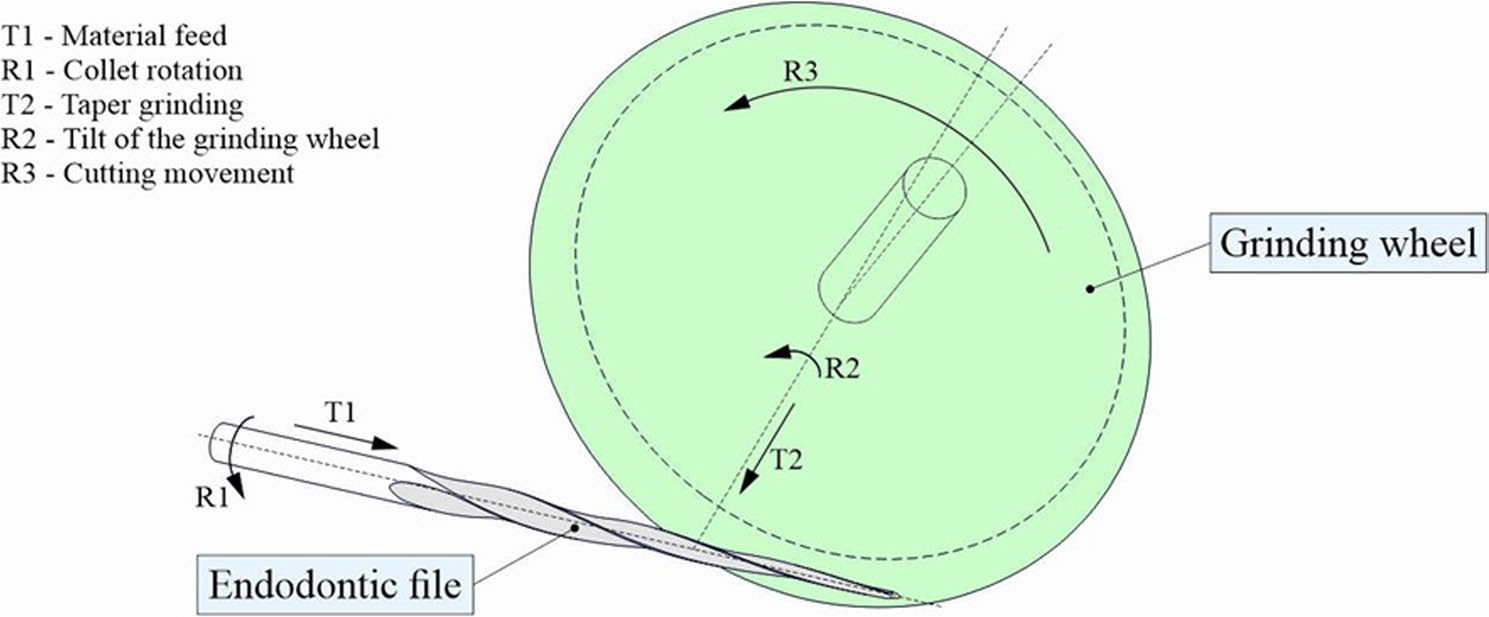
The endodontic file and grinding wheel

The computerized creation of the file model was accomplished at the convergence of two distinct geometries. The former was the raw material, whereas the latter was the machined (Fig. 2). The raw material corresponds to geometry of endodontic files before the cutting edges were machined over it.

Before the cutting edges were machined, the raw materials geometry matched that of the endodontic file. Its axisymmetric shape was made up of two separate parts: the active portion was represented by a truncated cone, and the shaft was represented by a cylindrical section.

**Fig. 3:**
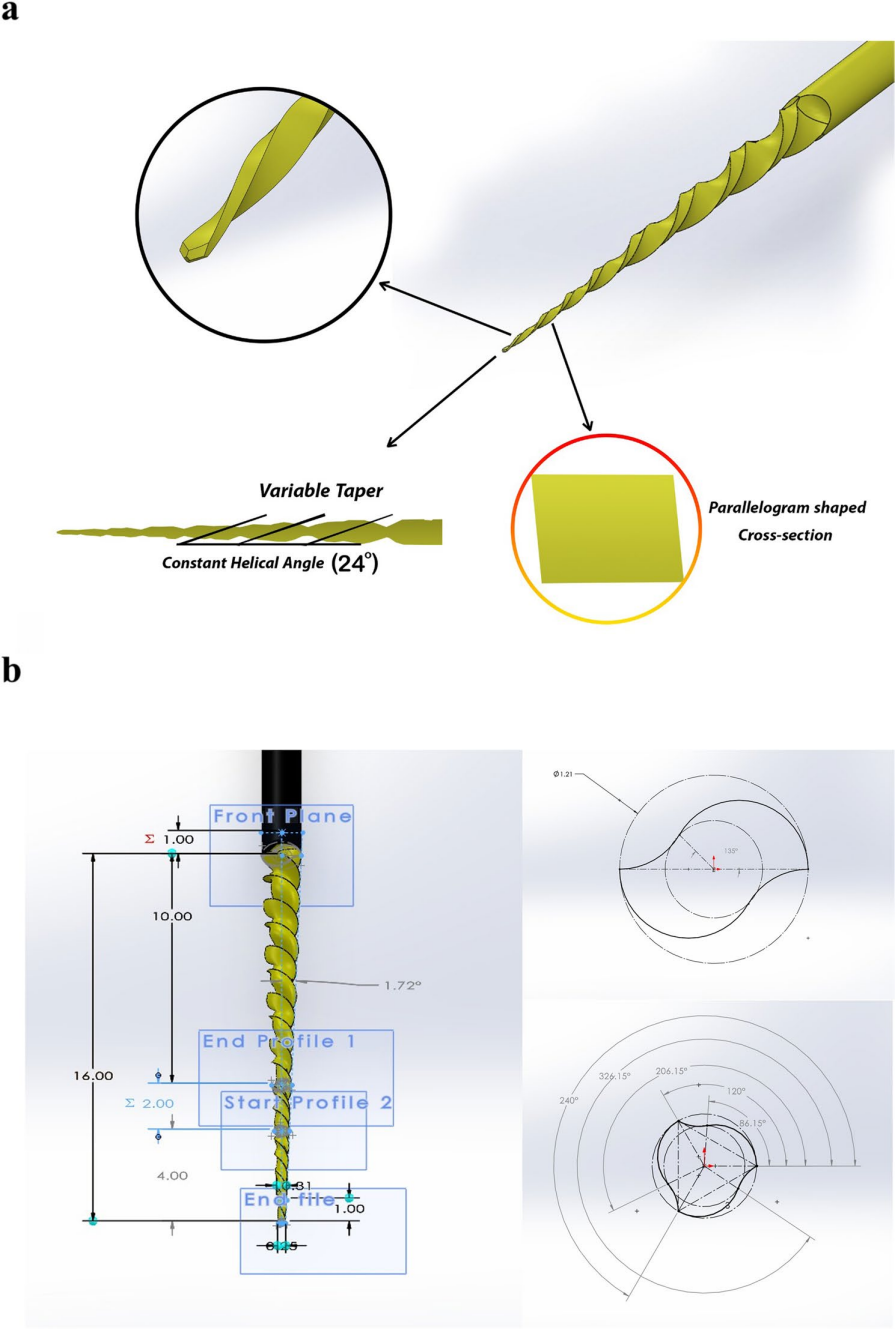
**a** Wave One Gold rotary file data 3d modeling, **b** One Curve rotary file data 3d modeling

To the raw materialized file, machining of blades along the length of active part was done taking into consideration the cross-section shape of two files. The final three dimension (3D) geometric model was obtained for Wave One Gold with the following dimensions: 25 mm in length, 16 mm in working surface, 7% taper, and 0.25 mm tip diameter, and One Curve, which was 25 mm long, had a tip diameter of 0.25 mm, a working surface of 16 mm, and a taper of 6% (Fig. 3)**.**

### Construction of 3D finite element model of root and simulated root canal curvatures

Schneider’s classification of root canal curvature had been considered to develop 3D root models with different degrees of canal curvatures. Three experimental models were designed in SolidWorks using Sketcher and Part Design modules having 2°, 30°, and 45° of canal angulations, representing straight, moderate, and severely curved canals. The 3D Finite model was constructed for a root length of 14 mm. The radius of curvature was kept constant at 4 mm for all the designed models. The thin layer of cementum covering the root was neglected, and the wall of the root was assumed to be dentin only. Mechanical properties of each part of the model were imported. The root canal orifice dimension was 0.30 mm, and the apical foramen diameter was assumed to be 0.13 mm.

One line with a length of 14 mm was drawn on a Sketcher Workbench. The second line was drawn intersecting the first line, assigning to the software the required degree of curvature. Using Lofted Bass in Part Design, 3D shape is generated.

A slot for the root canal space was created within the 3D model using the lofted cut tool with the presumed dimensions mentioned above. Based on the angle of curvature, the models built for the study were split into three groups: Group N: straight canals with 2° angulations; Group M: moderately curved canals with 30° angulation; and Group S: severely curved canals with 45° angulations.

### Defining the element type and mesh generation

**Fig. 4:**
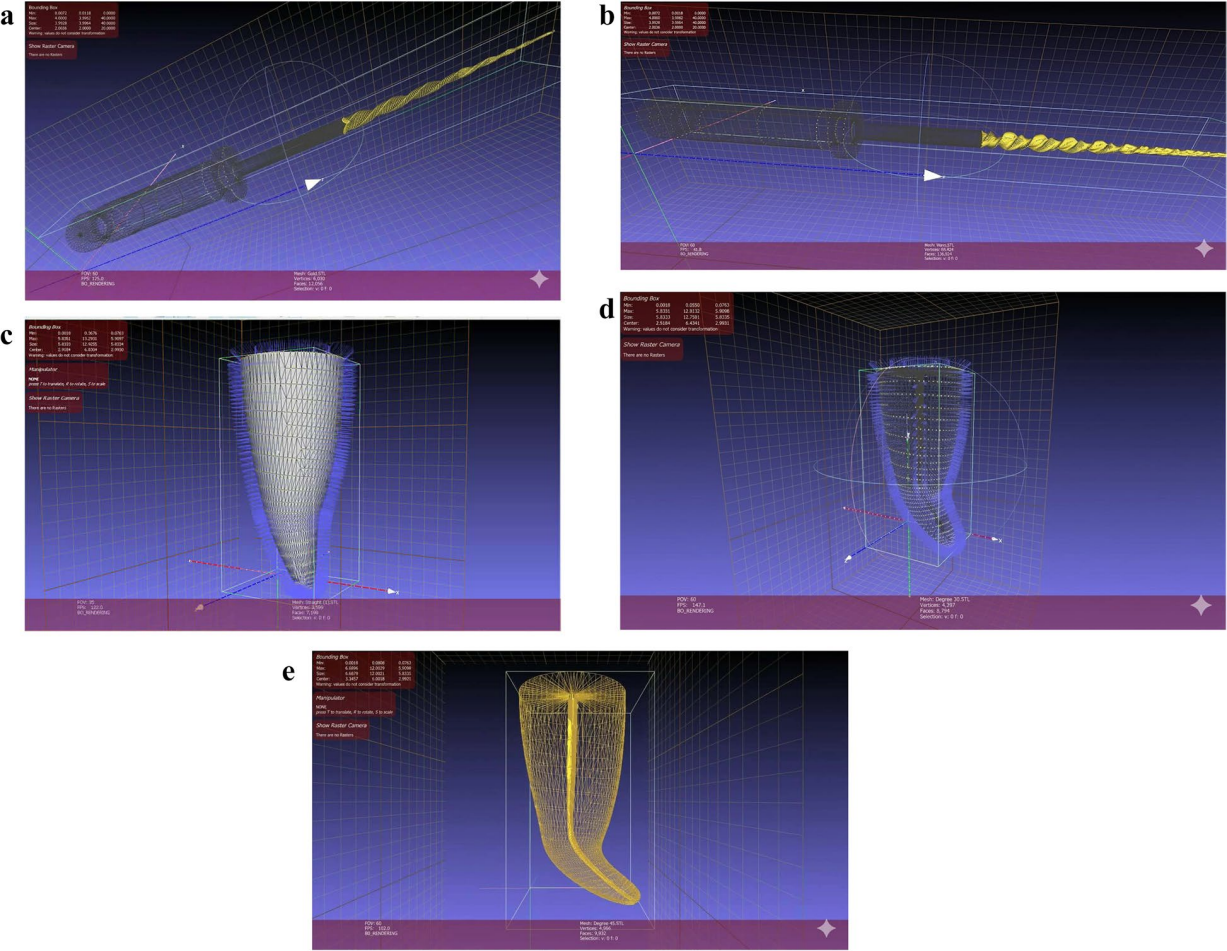
**a** Mesh generation of rotary file Wave One Gold, **b** Mesh generation of rotary file One Curve, **c** Mesh generation of tooth model straight, **d** Mesh generation of tooth model 30-degree curvature. **e** Mesh generation of tooth model 45-degree curvature

The studied Wave One Gold and One Curve endodontic files represented a defined parameterized geometry. Everyone had a cylindrical portion with diameter and a tapered helical part that was distinguished by its length, taper, constant or variable pitch, tip diameter, and cross-section form (Fig. 4)**.**

This stage required the definition of Wave One Gold, One Curve and tooth material properties, such as elasticity, Poisson’s ratio, density, and, if relevant, coefficient of expansion, friction, strength, etc… (Table 1).

**Table 1:** Properties of Ni Ti material [17]

Parameter	Description	Value
EA	Austenite elasticity	42,530 MPa
νA	Austenite Poisson’s ratio	0.33
EM	Martensite elasticity	12,828 MPa
νM	Martensite Poisson’s ratio	0.33
εL	Transformation strain	10%
(δσ/δT )L	(δσ/δT ) loading Start of	6,7
σL S	Transformation loading End	492 MPa
σL E	of transformation loading	630 MPa
T0	Reference temperature	22o C
(δσ/δT )U	(δσ/δT ) unloading Start of	6.7
σU S	Transformation unloading	192 MPa
σU E	End of transformation unloading	97 MPa
σME E	End of martensitic elastic regime	1200 MPa

For the Wave One Gold Primary and One Curve file models in the COMSOL software, extremely elastic Ni-Ti material was chosen. This material’s mechanical properties were described. The models were positioned with the file axis − z in the software. Wave One Gold simulation had 3294 elements and 6474 nodes, compared to 3155 elements and 7129 nodes in One Curve simulation. A mesh convergence test was performed to ensure that further mesh refinement did not significantly affect the calculated stress values. The selected mesh density represented an optimal balance between computational efficiency and numerical accuracy (Table 2)**.**

**Table 2:** Properties of tooth structures [18]

Material	Elasticity Modulus {E}	Poisson’s Ratio {*n*}
Dentin	18.6 Gpa	0.31
Periodontal Ligament	0.07 Gpa	0.45
Nickel titanium alloy	36 Gpa	0.3
Cementum	2.4 Gpa	0.3
Cortical bone	13.7 Gpa	0.30
High density Cancellous bone	1.37 Gpa	0.30

The model’s cross sections were shown as follows: The One Curve model’s cross-section geometry was off-center square, while the Wave One Gold model’s cross-section was off-center parallelogram.

Boundary conditions were used to measure the stress applied on One Curve and Wave One Gold files in different root canal curvatures (N, M, S). Throughout the file, at a working length of around 16 mm, boundary conditions were applied to each node in the section. The file was placed inside the root canal. Contact interaction between the instrument and dentin was defined within the finite element environment to simulate mechanical interaction during canal preparation. A simplified contact condition was assumed to allow stable numerical computation while maintaining realistic stress distribution; a virtual rotation of 250 rpm speed at all the curvatures was indicated. A torque of 2 N mm was applied to the long side of the file’s surface around the -z axis. To allow standardized comparison between the two file systems within the finite element environment, a controlled rotational motion was applied to both instruments during the simulation. This approach enabled consistent mechanical loading conditions across the tested models.

After preparing all required designs for stress analysis, two groups according to file system were categorised: either group Ⅰ Wave One Gold or group Ⅱ One Curve. Each group was designed according to canal curvature degrees (N, M, S), so group Ⅰ (Ⅰ N, Ⅰ M, Ⅰ S) and group Ⅱ (Ⅱ N, Ⅱ M, Ⅱ S). Von Mises stress values were noted on a colour map and represented numerically; the highest von Mises values were shown in red, while the lowest were shown in blue.

## Results

**Fig. 5:**
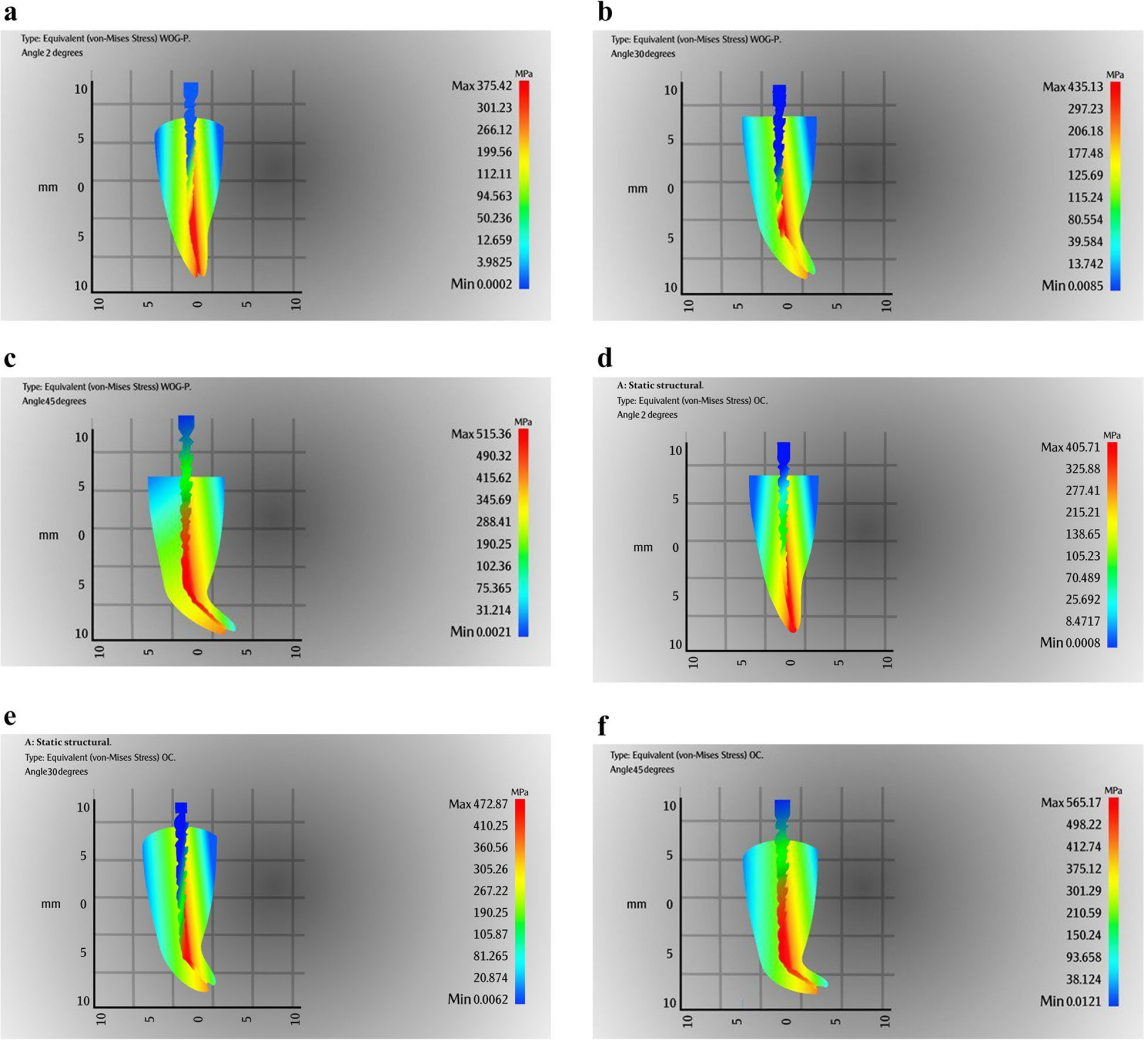
**a** Maximum von Mises stresses in group Ⅰ N, **b** Maximum von Mises stresses in Wave One Gold with 30-degree curvature in group Ⅰ M, **c** Maximum von Mises stresses in Group Ⅰ S, **d **Maximum von Mises stresses in group Ⅱ N, **e **Maximum von Mises stresses in group Ⅱ M, **f **Maximum von Mises stresses in group Ⅱ S

The Ni-Ti file’s mechanical behavior across all constitutive models was analyzed numerically in COMSOL Server 2020 software (Fig. 5).

In the present analysis, the least von mises stress values observed within GⅠ than GⅡ at the three different canal degrees (N, M,S) that exhibited 375.42 MPa at GⅠ N, 435.13 MPa at GⅠ M, and 515.36 MPa at GⅠ S.While 405.71 MPa at GⅡ N, 472.87 MPa at GⅡ M, and 565.17 MPa at GⅡ S (Table 3).

**Table 3:** Von Mises stresses of Wave One Gold and One Curve file systemes in N, M, and S canal curvatures

	Maximum von Mises stresses	
	GROUP Ⅰ (Wave One Gold)	GROUP Ⅱ (One Curve)
Straight 2^o^ (N)	375.42	405.71
Curvature 30^o^ (M)	435.13	472.87
Curvature 45^o^ (S)	515.36	565.17

## Discussion

One of the most frequent reasons for treatment failure in endodontic tooth management is instrument detachment. According to the American Association of Endodontics, the reported frequency rate for fractured rotary instruments lies in a range of 0.4% to 5% of cases [19, 20]. Broken rotary tool can be caused by a lot of variables, but the two main causes are torsional stress and cycle fatigue [21]. 

Torsional fractures happen when the tool’s tip locks into the channel while the head keeps rotating, exceeding the flexible threshold of torque material resistance. Repeated tensile pressure causes cyclic fatigue, which is most prevalent in curvy canals, where the tool is subjected to repeated stress and compression, especially at the maximum point of curvature [22]. Continuous advancements in the geometrical design and chemical composition of alloys have enhanced the mechanical performance of endodontic rotary files during operation [23]. 

Alloy’s composition, heat treatment, and instrument shape all influence the malleability of the NiTi instrument [24]. The NiTi alloy has three microstructure phases that are temperature-sensitive: austenite, martensite, and R-phase [25]. R-phase and martensitic NiTi are soft and flexible and have super-shape memory property, whereas austenitic NiTi is hard and robust [24]. 

Wave One Gold has improved flexibility due to its industrial advancement in gold-wire heat processing. The gold process is a post-manufacturing process that the ground NiTi files are heat-treated then gradually cooled. [26]. It revolves in a reciprocal rotation, with predetermined values by the company of clockwise/counterclockwise directions. Counter-clockwise, which is larger than the clockwise, permits the file to move apically, while the latter disconnects the file and removes file attachment [27]. This movement has a superior root preparation than continuous movement while minimising cyclic and torsional fatigue [28]. Compared with the continuous rotation of the endodontic files and regardless of other factors like degree of curve, rate of rotation, or material properties of the NiTi files, the reciprocating movement increases the resistance to cyclic and torsional fatigue [29]. 

A rotary system called One Curve is employed in a conventional continuous motion at 300 rpm with a torque of 205 N/cm that moves downward till the working length is reached [30]. It has a constant 6% taper, a changeable pitch, and an alternate cross-section (triple-helix toward its tip and S-section nearer its shank). It has been subjected to a NiTi heat process known as C Wire, which maintains the file in the martensitic phase and gives it a shape memory effect [31, 32]. 

Wave One Gold and One Curve are single endodontic files; since each file is not used for more canals and does not need to be thermally sterilised in an autoclave, it will not be subjected to additional stress. It simplifies instrumentation protocols to reduce stresses and to avoid the risk of cross-contamination [33, 34]. 

Comprehending all the technical features of both endodontic files and how they improve their function during preparation is very important to be chosen in the current investigation.

The simulated technique for evaluating stresses applied by root curvatures on Niti files has been induced by finite element (FE) models. To find, assess, and fix potential structural or performance issues, the FEA approach simulates a structure with loads and boundaries in a virtual setting [35, 36]. Previous studies demonstrated that numerical stress analysis can provide valuable information about the mechanical performance of endodontic instruments under different loading conditions and canal curvature furthermore, three-dimensional finite element models allow evaluation of forces and residual stresses generated during canal shaping, which may contribute to a better understanding of potential mechanical failure of NiTi rotary instruments [37–39]. 

COMSOL Multiphysics software was selected to be used in the current study as it is widely used to simulate biomechanical behavior to evaluate stresses using the von Mises criterion. The strength of COMSOL lies in its capability to model coupled phenomena, including chemical processes, heat transfer, fluid-structure interactions, and electromagnetics [40]. 

The von Mises stress is a number that indicates whether a material may fracture or yield. Metals and other ductile materials are its primary applications. It should be emphasized that von Mises stress represents an indicator of instantaneous stress distribution within the material and does not directly predict cyclic fatigue life or the number of cycles to fracture [41, 42]. Colour coded map resulted that illustrated the variability of stress values between groups and in different parts of the models.

Those findings reject the null hypothesis, thus there was a difference in stress values between the two-file systems in the studied canal curvatures. In addition, there was an increase in stress values as the angulation of curvature increased.

Previous studies have demonstrated that reciprocating motion may influence the cyclic fatigue resistance of nickel–titanium instruments when compared with continuous rotary motion [43]. For optimal performance in intricate curvatures and root canal anatomy, gold-wire heat treatment is used to keep the file in the martensitic phase during clinical treatment [43]. These findings were consistent with those of Siva et al., who found that, when compared to M-wire instruments, all gold heat-treated files had improved flexibility and fatigue resistance [44]. 

The results of this study supported the findings of Ozyurek et al. [44] who claimed that the cyclic fatigue resistance of the Wave One Gold Primary, which was formed by gold-wire heat processing, had stronger cyclic fatigue resistance than Wave One Primary and Reciproc R25(VDW, Germany) which both were manufactured from M-wire due to its 2-stage transformation behaviour, although three of them were reciprocating endodontic rotary files.

Wave One Gold showed less stress values than One Curve indicating differences in stress concentration patterns between the two instrument systems, and this was in compliance with Merima et al., [45] Bueno et al. [46] Alcalde et al., [47] Castello-Escriva et al. [48], and Kiefner et al. [49] studies, they showed that reciprocating motion improved endodontic file’s resilience to cyclic fatigue, reduced the likelihood of instrument breakage within the root canal, and enabled improved debris removal compared to continuous rotation. However, these results were contrary to Gundogar et al. [50] and Oh et al., [51] who determined that HyFlex EDM’s(Coltene/Whaledent, Altstätten, Switzerland) rotating motion demonstrated the highest cyclic fatigue resistance after comparing the cyclic fatigue resistance of Wave One Gold, Reciproc, and Hyflex EDM. In contrast to the One Curve file which subjected to continuous rotation, it experienced unidirectional torque, which caused the file to be under continual stress and strain Subhashini N et al. Because of the dentin’s hardness and the canal’s curve, it puts the file under constant, fluctuating pressure [52]. 

Wave One Gold has a parallelogram cross section vs. One Curve, which has a triple-helix section toward its tip and an S-section nearer its shank. A patented off-centred cross-section, in which only one cutting edge is in contact with the canal wall, reduces the screw in force by decreasing the instrument’s contacts with the tooth’s canal wall while simultaneously increasing the space required for debris removal. This is why Wave One Gold demonstrated less stress [53]. 

According to Xu & Zheng [54] the cross section of the instrument was a crucial factor in stress concentration. Wave One Gold, which had a parallelogram cross section, possessed a similar reciprocating action to Wave One, which had a convex triangular cross section, which improved its physical attributes, including 23% more efficacy, 50% greater resistance to cyclic fatigue, and 80% greater flexibility than Wave One [55, 56]. 

The Wave One Gold file’s maximum stress was focused on the tip and diminished as it went away. With a constant apical 3 mm, Wave One Gold Primary and One Curve both had a tip size of 25 and taper sizes of 0.07 and 0.06, respectively. As a result, Wave One Gold’s larger apical taper produced a higher torsional than the One Curve. File size and torsional resistance were directly correlated, according to Ninan & Berzins. Similarly, instruments with a larger taper showed more torque but a smaller rotational angle [57]. 

Among the studied groups, an increase in curvature angle resulted in higher stress values along the instruments. According to earlier research, the radius and angle of the root canal curvature had a significant impact on the file’s cyclic fatigue resistance [58, 59]. This result was explained that any point of a spinning file inside a curved root canal experiences alternating compressive and tensile loads and strains [60]. According to Sattapan et al., [61] Shen et al., [62] Spanaki-Voreadi et al. [63] and Cheung et al. [64] fatigue was the primary cause of file breakage clinically. Even so, the various root curves continuously render the root canal preparation difficult, which jeopardises the effectiveness of endodontic treatment [65]. 

From a clinical perspective, lower stress concentration within an instrument may reduce the likelihood of mechanical failure during canal preparation, particularly in severely curved canals. Therefore, instruments demonstrating lower stress levels under simulated curvature conditions may present a potential advantage in maintaining mechanical integrity during clinical use. Nevertheless, the clinical performance of endodontic instruments is influenced by multiple additional factors including operator technique, canal anatomy, and dynamic loading conditions.

Despite the advantages of finite element analysis in evaluating biomechanical behavior, several limitations must be acknowledged. FEA studies are generally virtual in nature and the direct application of their results in the clinical practice is somewhat limited. Moreover, the simulated models cannot fully reproduce the complex clinical conditions encountered during endodontic treatment, including variations in dentin properties, anatomical irregularities, irrigation dynamics, and operator-related factors. Additionally, the simplified loading conditions used in the present study represent an approximation of clinical instrumentation mechanics.

## Conclusion

Within the limitations of this finite element analysis study, Wave One Gold demonstrated lower von Mises stress values compared with One Curve under different canal curvature conditions. These findings may be related to differences in instrument design, alloy metallurgy, and kinematics of motion. In addition, increasing canal curvature resulted in higher stress values for both file systems. Although the observed differences suggest variations in mechanical behavior, caution should be taken when extrapolating these results directly to clinical conditions. Therefore, further experimental and clinical studies are recommended to confirm the clinical relevance of these findings.

## Data Availability

Data generated are available upon request from the corresponding author.
